# pBAM1: an all-synthetic genetic tool for analysis and construction of complex bacterial phenotypes

**DOI:** 10.1186/1471-2180-11-38

**Published:** 2011-02-22

**Authors:** Esteban Martínez-García, Belén Calles, Miguel Arévalo-Rodríguez, Víctor de Lorenzo

**Affiliations:** 1 Systems Biology Program. Centro Nacional de Biotecnología-CSIC (Calle Darwin 3, Campus de Cantoblanco), Madrid (E-28049), Spain; 2 Biomedal SL (Av. Américo Vespucio, 5), Sevilla (E-41092), Spain

## Abstract

**Background:**

Since publication in 1977 of plasmid pBR322, many breakthroughs in Biology have depended on increasingly sophisticated vector platforms for analysis and engineering of given bacterial strains. Although restriction sites impose a certain format in the procedures for assembling cloned genes, every attempt thus far to standardize vector architecture and nomenclature has ended up in failure. While this state of affairs may still be tolerable for traditional one-at-a-time studies of single genes, the onset of systems and synthetic biology calls for a simplification -along with an optimization- of the currently unwieldy pool of genetic tools.

**Results:**

The functional DNA sequences present in the natural bacterial transposon Tn*5 *have been methodically edited and refactored for the production of a multi-purpose genetic tool named pBAM1, which allows a range of manipulations in the genome of Gram-negative bacteria. This all-synthetic construct enhances the power of mini-transposon vectors for either de-construction or re-construction of phenotypes *á la carte *by incorporating features inspired in systems engineering: modularity, re-usability, minimization, and compatibility with other genetic tools. pBAM1 bears an streamlined, restriction site-freed and narrow-host range replication frame bearing the sequences of R6K *oriV*, *oriT *and an ampicillin resistance marker. These go along with a business module that contains a host-independent and hyperactive transposition platform for *in vivo *or *in vitro *insertion of desired DNA into the genome of the target bacterium. All functional sequences were standardized for a straightforward replacement by equivalent counterparts, if required. pBAM1 can be delivered into recipient cells by either mating or electroporation, producing transposon insertion frequencies of 1.8 × 10^-3 ^and 1.02 × 10^-7^, respectively in the soil bacterium *Pseudomonas putida*. Analyses of the resulting clones revealed a 100% of unique transposition events and virtually no-cointegration of the donor plasmid within the target genome.

**Conclusions:**

This work reports the design and performance of an all-synthetic mini-transposon vector. The power of the new system for both identification of new functions or for the construction of desired phenotypes is shown in a genetic survey of hyper-expressed proteins and regulatory elements that influence the expression of the σ^54^-dependent *Pu *promoter of *P. putida*.

## Background

The issue of modularity in genetic constructs has been present in the microbiological literature since the onset of recombinant DNA [1]. Despite various attempts to format vector structure and nomenclature [2], there is not yet any generally accepted standard for plasmid architecture or physical assembly of cloned DNA sequences. This state of affairs is rapidly becoming a bottleneck as we move from handling just a few genes in typical laboratory organisms into analysing and massively refactoring the genomes of very diverse bacteria. The notion of formatted genetic tools for the analysis and stable engineering of microorganisms was pursued in the early 90s (among others) with the design of the so-called mini-transposon vectors [3]. These allowed stable insertions of foreign DNA into the chromosome of virtually any Gram-negative target. Tn*5*-derived constructs presented a large number of advantages over their plasmid-based counterparts for introduction of transgenes into many types of bacteria [3-5]. These included maintenance without antibiotic selection, long-term stability and re-usability for generating multiple insertions in the same cells, with no apparent size limits. Yet, the original design of such mini-transposons [4,5] was plagued with problems, such as the inheritance of long, non-functional DNA fragments carried along by the intricate cloning-and-pasting DNA methods of the time. These were also afflicted by the excessive and inconvenient number of non-useful restriction sites scattered along the vectors, and the suboptimal transposition machinery encoded in them. Despite downsides, the mini-transposon-bearing pUT plasmid series [3] are still to this day one of the most popular vector platforms for analysis and engineering of Gram-negative bacteria. In fact, every successful feature of the classical mini-Tn*5*s and its delivery system is originated in mobile elements (broad host range plasmids and transposons), which are naturally evolved to thrive in a large variety of hosts. In particular, the Tn*5 *transposition system requires exclusively the transposase encoded by *tnpA*, and the terminal ends of the transposon as the substrate. This affords transposition in a fashion virtually independent of the host, thereby qualifying as an orthogonal biological machinery that expands the utility of the vectors to virtually any host [6].

In this work we have exploited the current ease of DNA synthesis for a dramatic remake of the original mini-Tn*5 *transposon vector concept. In this process, the most attractive features have been enhanced while each of the drawbacks (identified along 20 years of use in hundreds of laboratories) has been eliminated. To this end, we have revisited the functional modules that shape the vector and have edited the corresponding DNA sequences to minimize them, improve their functionality and make them entirely modular and exchangeable. The final product was the entirely synthetic construct that we have named pBAM1 (for **b**orn-**a**gain **m**ini-transposon), which multiplies the benefits of the earlier designs. We show below that this genetic tool is most advantageous not only for random mutagenesis studies on a target bacterium such as *Pseudomonas putida*, but also for implantation of functional *cargos *into its genome, be they one (or few) transgene(s), a transcriptional reporter, or a complex genetic or metabolic circuit. The applications are illustrated below in two different contexts. One regards the identification of new functions that influence the regulation of the catabolic σ^54^-dependent *Pu *promoter of *P. putida*. The other involves the visualization of the intracellular targeting of highly expressed proteins in individual bacteria by means of random generation of GFP protein fusions.

## Results and Discussion

### Rationale of the pBAM1 layout and editing of its functional modules

**Figure 1:**
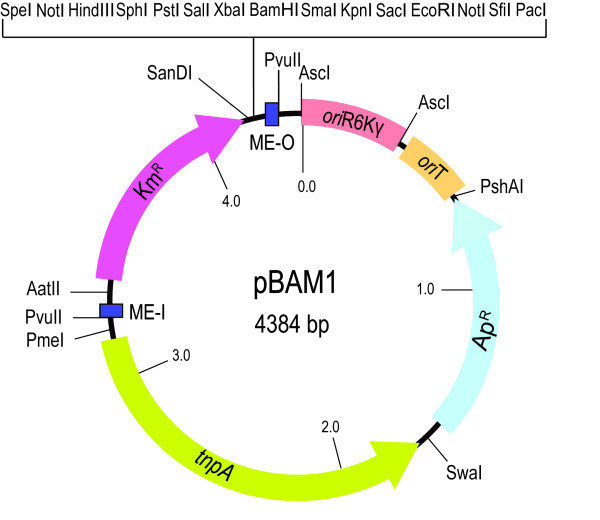
**pBAM1 plasmid map**. Functional elements of the plasmid include relevant restriction sites, antibiotic markers (Ap, ampicillin, Km, kanamycin), transposase (*tnpA*), origin of replication (R6K), the origin of transfer region (*ori*T), mosaic element O (ME-O), and mosaic element I (ME-I), as shown.

A map of the pBAM1 plasmid is shown in Figure 1, with an indication of all functional modules assembled in a total of 4384 bp of synthetic DNA. The complete sequence can be retrieved from GenBank with the accession number HQ908071. The serviceable DNA segments included in the construct and the implementation of enhanced properties in each of them are separately examined below. They include the plasmid frame (which embodies a system for suicide delivery to potential recipients), the mini-transposon element and the cargo module.

The first key feature of pBAM1 is the utilization of the narrow host-range origin of replication of plasmid R6K as the vegetative *oriV *of the construct for its proliferation. This origin is strictly dependent on the so-called π protein (encoded by the *pir *gene of R6K). The *oriV *and the *pir *gene of R6K can be separated and made to function in *trans *[7]. This makes replication of any covalently close circular (ccc) DNA bearing such an *oriV *entirely dependent on the provision of the p protein, either from a second plasmid or from the chromosome. This feature has been exploited for the development of a number of conditional systems that make replication of a given construct addicted to host strains of *E. coli *that express the *pir *gene [8]. Virtually all of such existing systems carry the R6K*oriV*-containing 420 bp fragment from pGP704 plasmid [8]. This naturally occurring DNA sequence is not only longer than necessary for the *oriV *function, but it also carries an internal HindIII site in the midst of the repeats that are recognized by the replication machinery [9,10]. Moreover, this segment in pGP704 has flanking EcoRI and BamHI sequences that prevent the cognate restriction enzymes being used for cloning. For pBAM1, the whole *oriV *region was streamlined to a minimum (392 bp) and the internal HindIII removed (while keeping a sequence in the former site with similarity to the functional repeats). Finally, the termini of the segment were furnished by the infrequent restriction site AscI to create the origin of replication module. These changes did not affect any of the properties described for the natural R6K*oriV *sequences [9]. pBAM1 and its derivatives are maintained in the specialized strain *E. coli *CC118λ*pir*, which expresses the π protein from a lysogenic phage [4].

The next module of the plasmid frame was the sequence that contains the origin of transfer *oriT *(Figure 1) and enables transfer of pBAM1 from the host strain to a new recipient, when recognized by the conjugative machinery encoded by the broad host range plasmid RK2, also called RP4 [11]. Since the RP4/RK2 conjugal transfer system is the most promiscuous of all DNA mobilization device known, the presence of *oriT *allows mobilization of pBAM1 into virtually any Gram-negative or Gram-positive bacteria [12] and can even be passed into fungi [13] and eukaryotic cells [14], provided that the construct is exposed to the action of the Tra proteins of RP4 [8]. This transfer can be made by either setting up a tri-parental mating mixture with a donor strain (e.g. *E. coli *CC118λ*pir*) bearing the R6K*oriV/*RP4*oriT *plasmid, a recipient bacterium and helper cells bearing the *mob*/*tra *region of RP4 cloned in a plasmid which does not replicate in the recipient [8]. As an alternative, the donor λ*pir*^+^ strain may have the *tra/mob *functions integrated in its chromosome (for instance, *E. coli *S17-1λ*pir*) allowing bi-parental mating [15]. Other λ*pir*^+^*E. coli *donor strains such as *E. coli *RH03, which have been engineered to facilitate counter-selection, are also eligible to this end [16]. The *oriT *region employed in most plasmid vectors designed for mobilization purposes is exceedingly large (1728 bp) and flanked by BamHI sites [8]. As before, we trimmed down the *oriT *to the minimum of 244 bp required for functionality [11], eradicated one SfiI site present within the core *oriT *sequence (to allow its inclusion in the polylinker of the vector) and the streamlined module was flanked by the two rare enzyme sites FseI and PshAI. Note, however, that in some cases the plasmid can just be electroporated into target cells and conjugation may not be necessary, although the efficiency is considerably lower. Since the plasmid transferred to the recipient by conjugation or electroporation cannot in any case replicate because of the lack of the p protein, this process is called *suicide delivery*.

The last module of the plasmid frame of pBAM1 was the *bla *gene that encodes a β-lactamase, endowing Ap resistance as selective marker. We kept the natural P3 promoter of the natural *bla *gene to control its expression; [17] and maintained the protein sequence of the enzyme that is employed by many other vectors [18], but the codon usage of the gene was optimized for *E. coli *and potentially conflicting restriction sites removed. Furthermore, transcriptional terminators from the *trpA *gene (alpha subunit of the tryptophan synthase from *E. coli*) and the gene VIII from phage fd were placed upstream and downstream of the *bla *gene, respectively, to avoid transcriptional readthrough from neighbouring sequences. Finally, this selection marker module was flanked by SwaI and PshAI sites, as shown in Figure 1.

Next come the elements engineered in pBAM1 for causing insertions of cloned DNA into the genome of the target strain. These include a segment encoding the transposase gene *tnpA *lying outside but adjacent to a DNA segment flanked by the terminal sequences of Tn*5 *(i.e. the mini-transposon itself). The Tn*5 *transposase recognizes both end-sequences and catalyzes the transfer of the mobile module from the donor replicon to the target genome, where it randomly inserts (there is a slight preference for G/C at both ends of the 9-bp target sequence; [19]). The configuration in pBAM1 exploits the fact that the Tn*5*-carried *tnpA *gene also works well when located outside the mobile element, although it still needs to be in *cis *in respect to the target sequences of its gene product [20,21]. The sequence of the Tn*5 tnpA *gene of pBAM1 was edited to enhance a number of desirable characteristics. First, instead of the naturally occurring gene, which has evolved to mediate a very low level of transposition, we re-designed *tnpA *to endow its product with hyperactivity [22]. This included an E54K substitution, which increases transposase binding to the terminal OE sequence, a M56A change that blocks the synthesis of the Inh protein (a trans-dominant negative truncation of TnpA that represses transposition), and a L372P replacement that enhances TnpA dimerization, thereby improving its activity [22]. As before, to eliminate inconvenient restriction sites, the NotI sequence indigenous to the IS*50*R part of Tn*5 *was removed by a silent substitution G_504_->C [4]. In addition, the *tnpA *stop codon TGA was changed by the more efficient TAA termination codon. Otherwise, the edited transposase gene was expressed through its natural T1 promoter. However, as *tnpA *expression is downregulated by methylation, the two *dam *recognition sites (5'-GATC-GATC-3') present within this promoter region were changed to 5'-AATC-GATG-3' as described [23]. The sum of all these operations yielded an optimized transposase variant carried by a 1524 bp segment flanked by PmeI and SwaI sites.

**Figure 2:**
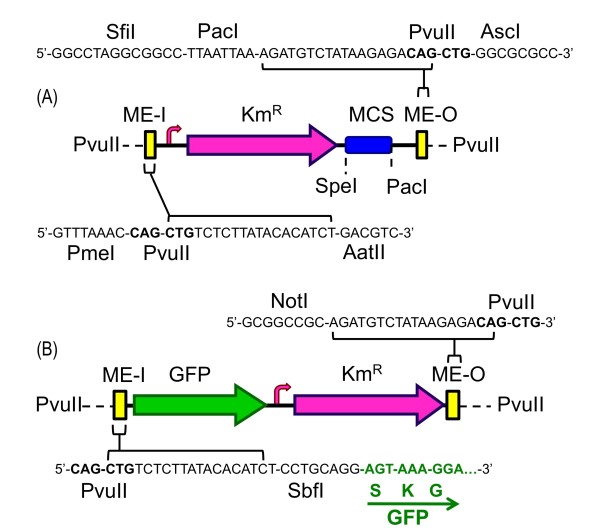
**Structural organization of standard mini-transposon modules**. **(A) **Mini-Tn*5 *Km. Details of relevant restriction enzymes within the module are shown. The fusion of ME-I and ME-O sequences with the plasmid DNA backbone generated PvuII restriction sites that bracket the mobile segment. The red arrow indicates the position of the promoter of the Km resistance gene. MCS: multiple-cloning-site. (**B) **mini-Tn*5*GFPKm. Schematic representation of the main features of this version of the mini-transposon engineered in the pBAM1 backbone, containing the GFP gene lacking leading sequences and thus able to produce protein fusions upon chromosomal insertions in the right direction and frame. The Km resistance cassette is identical to that of the mini-Tn*5*Km of pBAM1.

All the features were assembled for the only purpose of assisting the performance of the functional module of pBAM1: the mini-transposon segment, the boundaries of which are its two terminal end-sequences (ES). Composite transposons like Tn*5 *have two full insertion sequence (IS) elements at their termini; both of IS sequences are similar but not identical bracketed by 19-bp ESs known as inside (IE) and outside (OE) end, which are specifically bound by the transposase [6]. In its natural context, TnpA can bind the OE and IE of IS*50*s and promote transposition of only one insertion sequence. Alternatively, the same protein can bind the outer OEs of the whole transposon and provoke transposition of the entire Tn*5 *[6,24]. Instead of such natural arrangement, we flanked the mini-transposon part of pBAM1 with the optimized and hyperactive 19-bp mosaic sequence (ME) previously characterized [25]. These were designated ME-I and ME-O to determine the orientation within the plasmid frame, but are identical in sequence. Note that the external borders of both MEs were endowed with unique PvuII restriction sites (Figure 2), thereby allowing the excision of the mini-transposon as a linear, blunt-ended DNA which can be combined with a purified transposase to form a transposome for its *in vivo *[26] or *in vitro *[22] delivery to a target DNA.

Although a large number of useful sequences can be placed between ME-I and ME-O, the mini-transposon carried by pBAM1 carries a Km resistance gene (*neo*) from Tn*903 *as a default selection marker, as well as what we call a *cargo site *containing a polylinker for general cloning purposes. As before, the natural *neo *sequence (GenBank: V00359; [27] was edited to improve codon usage and to eliminate the naturally occurring SmaI and HindIII sites at positions 306 and 550 respectively from the start codon of the *neo *gene. The resistance gene was expressed through its natural, broad host range promoter, which spans 81 bp upstream of the start codon of the *neo *gene, the entire Km^R ^cassette being bracketed by terminal AatII and SanDI restriction sites. These anchor the *neo *gene within the transposable segment of pBAM1 and allow its replacement when required by other selectable markers. The cargo site consists of a polylinker for thirteen unique restriction sites flanked with sites for the rare cutter enzyme sites SpeI and PacI. One site (NotI) is however repeated at both ends of the polylinker, because its internal deletion reconstructs a short NotI-SfiI sequence that makes it compatible with earlier versions of mini-transposons [4,5]. In contrast to these, however, the cloning sites of the polylinker are unique in pBAM1, making unnecessary the two-step cloning protocols that afflicted the former chromosomal insertion strategies [15]. The final assembly thus has the start codon of the *neo *gene 107 bp downstream of the ME-I, while the stop codon is 174 bp downstream of the ME-O, the total length of the optimized element becoming 1135 bp (Figure 2A).

The modular layout of the functional segments of pBAM1 allows the replacement of each of them by equivalent counterparts, leaving intact the others. We thus argue that the rare sites that punctuate the structure of the vector (Figure 1) provide a useful standard for physical assembly of equivalent systems with other origins of replication, other transposable systems e.g. mariner [28], Tn*7 *[29], and other selection markers. Once the study of each module was made along the lines mentioned above and the sequences edited *in silico*, the whole was assembled to produce a unique sequence of 4384 bp that was chemically synthesized.

### Validation of pBAM1

To assess the functionality and versatility of the new synthetic vector we passed it through several experimental tests to check that the plasmid and the new minimized standard features worked as expected. First we verified that the construct was stably propagated in *E. coli *CC118*λpir*, as a medium-to-high copy number plasmid (not shown). This confirmed that the editing of the HindIII site in one of the repeats of R6K*oriV *previously believed to be critical for replication [9] was tolerated by the plasmid without any detrimental effect. We next tested two different methods for suicide delivery of the plasmid into a recipient strain (*P. putida *KT2440), which is a good representative of the non-enteric Gram-negative bacteria widely used in industrial and environmental microbiology [30-32]. First, we employed a standard tri-parental mating (see Materials and Methods) for verifying the transposition process and determining the optimum period of time required for constructing a saturated transposition insertion library. To this end, the mating mix was allowed to conjugate for 1 to 18 h on filters laid on LB plates. At the times indicated, the cells on the filters were resuspended and plated onto M9-citrate agar with Km for removal of the donors and selection of *P. putida *clones bearing insertions of the mini-Tn*5 *element. As shown in Additional File 1 (Figure S1), the average frequency of Km^R ^exconjugants ranged from 0.006 ± 0.008 × 10^-3 ^after one hour of mating, to 6.2 ± 0.15 × 10^-3 ^at eighteen hours. The number of exconjugants at longer times, although higher, were not considered as they surely reflected the amplification of earlier transposition times through cellular division, instead of new transposition events. In view of the bimodal shape of the time course of Figure S1 (see Additional File 1) we picked 5 h as the most useful time for maximum conjugation/transposition events with a minimum of growth. The next step was to examine whether exconjugants had undergone authentic transposition events or they resulted from the cointegration of pBAM1 into the host genome. 200 colonies were randomly selected and their sensitivity to the plasmid marker (Ap^R^) tested. All 200 Km^R ^clones turned out to be sensitive to the β-lactam antibiotic ampicillin (500 μg ml^-1^), thereby indicating that the insertion of the mini-transposon carried by pBAM1 had occurred as expected.

In view of the high numbers, we wondered whether pBAM1 could also be delivered to *P. putida *cells in a suicide manner through electroporation instead of conjugation. Given that the plasmid cannot replicate in the recipient (see above) this method has the potential advantage that every Km^R ^colony developed on the selective plate must come from a unique transposition event and that siblings are then avoided. Table 1 shows that, despite being less efficient than conjugation, transformation of pBAM1 did result in a large number of Km^R ^clones, in a dose-dependent fashion with regards to the amount of DNA entered in the transformation mixture. As before, all of 100 such Km^R ^colonies tested were sensitive to Ap, as they resulted from *bona fide *transpositions, rather than co-integration of the donor plasmid into the target genome.

**Table 1:** Transposition frequencies of pBAM1

	Resistance frequency		Analyses of exconjugants		
**Technique^a^**	**Spontaneous^b^**	**Non-spontaneous^c^**	**Sample^d^**	**Transposition^e^**	**Cointegrates^f^**

Mating	Not detectable	1.8 ± 0.53 × 10^-3^	200	200	0
Electroporation	Not detectable	1.02 ± 0.38 × 10^-7^	100	100	0

^a ^The pBAM1 plasmid was introduced into recipient cells either by five-hour tri-parental mating or by electroporation, letting the cells to recover after the electro-pulse in LB at 30°C for one hour. Electroporation figures are the average of the frequencies obtained using 100 ng (1.1 ± 0.5 × 10^-7^) and 500 ng (0.89 ± 0.2 × 10^-7^) of plasmid DNA.

^b ^Number of *P. putida *KT2440 colonies that acquire the marker resistance spontaneously, without mating or electroporation.

^c ^Total number of cells that acquired the mini-transposon, as measured by growth in kanamycin normalized to the total 3 × 10^7 ^donor cells. The 5 h mating frequency was averaged using a total of 16 independent experiments. Electroporation was referred to a final cell concentration of 6 × 10^10 ^electrocompetent cells and the frequency determined with 6 independent experiments.

^d ^Number of independent colonies that were screened for the presence of the mini-transposon marker (kanamycin) and for the loss of the plasmid backbone marker (ampicillin).

^e ^Number of kanamycin resistant colonies.

^f ^Colonies that are both resistance to kanamycin and ampicillin, meaning co-integration of the pBAM1 plasmid into their genome.

The next step in the validation involved assessment of the randomness of insertions, the possible occurrence of multiple transposition events in the same cell, and the degree of saturation of each gene with the mobile element. A first answer to these questions was provided by the precise mapping of the boundaries of the mini-Tn*5 *insert in one dozen randomly picked Km^R ^colonies coming from either procedure. To this end, we employed the PCR method of Das *et al *[33] with arbitrary primers ARB6 and ARB2 (Table 2) along with a second set of cognate primers that hybridize either end of the mini-transposon (ME-I and ME-O, Table 2). For determining the site of insertion of the transposons we employed in each case primer sets for both ends (ME-I and ME-O). Figure S2 (Additional File 1) shows just one example of using this strategy for mapping the mini-Tn*5 *insertions at the ME-O end with arbitrary PCR. The twenty-four sequences yielded similar results that allowed both to locate insertions within the genome of *P. putida *and to rule out double or multiple transposition events (Additional File 1, Table S1). 9 out of the 12 insertions occurred in structural genes scattered through the genome whereas 3 of them ended up within intergenic regions. The sequencing of a good number of transpositions of the mini-Tn*5 *element born by pBAM1 (and its variant pBAM1-GFP) allowed us to examine possible biases of the mobile element for specific sequences. Analysis of fifty-five 9-bp of the host genome duplicated after mini-Tn*5 *insertion [6] revealed that this was not the case (Additional File 1, Figure S3) and that insertion of the synthetic mini-transposon(s) was virtually random.

**Table 2:** Primers used in this study

Name	Sequence 5' → 3'	Usage	Reference
ARB6	GGCACGCGTCGACTAGTACNNNNNNNNNNACGCC	PCR round 1	[59]
ARB2	GGCACGCGTCGACTAGTAC	PCR round 2	[59]
ME-O-extF	CGGTTTACAAGCATAACTAGTGCGGC	PCR round 1	This work
ME-O-intF	AGAGGATCCCCGGGTACCGAGCTCG	PCR round 2/sequencing	This work
ME-I-extR	CTCGTTTCACGCTGAATATGGCTC	PCR round 1	This work
ME-I-intR	CAGTTTTATTGTTCATGATGATATA	PCR round 2/sequencing	This work
GFP-extR	GGGTAAGTTTTCCGTATGTTGCATC	PCR round 1	This work
GFP-intR	GCCCATTAACATCACCATCTAATTC	PCR round 2/sequencing	This work

**Figure 3:**
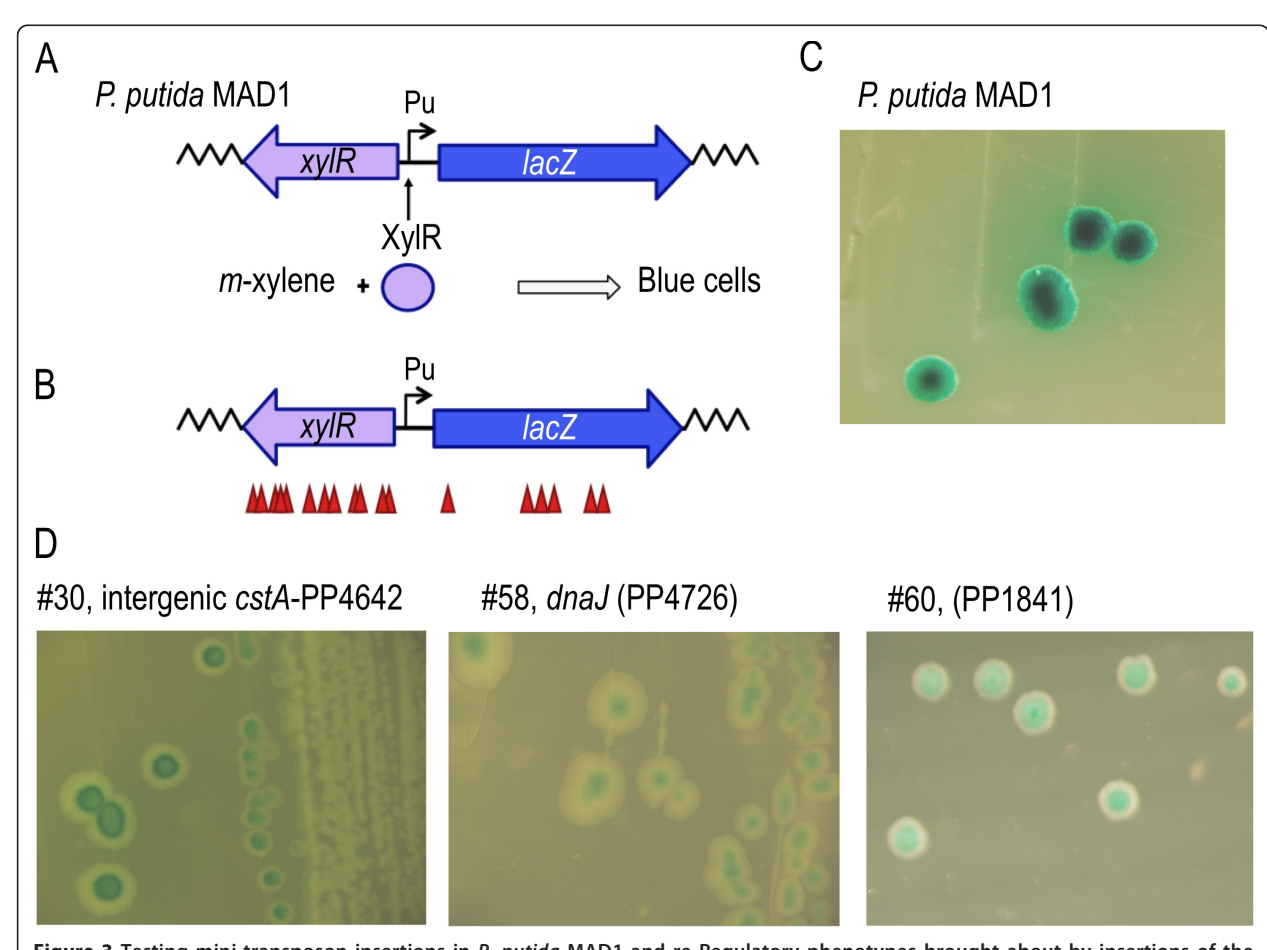
**Testing mini-transposon insertions in *P. putida *MAD1 and re Regulatory phenotypes brought about by insertions of the mini-Tn*5*Km of pBAM1 in *P. putida *MAD1**. **(A) **Representation of the reporter module born by the *P. putida *MAD1 strain. *Pu *is induced by XylR in the presence of *m*-xylene vapours. (**B**) Schematic representation and approximate location of mini-Tn*5*Km insertions within *xylR *and *lacZ *in *P. putida *MAD1. **(C) **The reference condition is that of the clones of the non-mutagenized strain exposed to *m-*xylene and grown on a plate with X-gal for several days, which results in an intense blue colour exacerbated in the centre of the colony. **(D) **The other pictures represent the variety of the blue/white patterns obtained throughout the *P. putida *MAD1 mutagenesis experiment. The pictures were obtained with a Leica MZ FLIII stereomicroscope with an Olympus DP70 camera. See Table S3 of Additional File 1 for more details.

To obtain a more accurate measurement of the frequencies and diversity of insertions, we employed a strategy that relied on the appearance of a known visual phenotype. For this, we used a derivative of *P. putida *KT2442 strain called *P. putida *MAD1, which bears in its chromosome an *m-*xylene responsive *Pu*-*lacZ *transcriptional fusion that is activated by the σ^54^-dependent protein XylR, which is encoded also in its genome (Figure 3A; [34]) The *Pu *promoter has a very low basal expression level but becomes strongly activated when *P. putida *MAD1 is exposed to *m*-xylene and yields blue colonies. The loss of any of 3 genetic elements of the strain (*lacZ*, XylR and σ^54^, encoded by *rpoN*) can thus yield white colonies on a background of blue clones when exposed to *m-*xylene vapours. The incidence of insertions in each of the genes can accordingly provide a good estimation of the global transposition frequency. To tackle this question, *P. putida *MAD1 strain was mutagenized by tri-parental mating, plated on a minimal M9 citrate-Km medium supplemented with Xgal, and the Km^R ^colonies subject to saturating *m*-xylene vapors. 18 out of the thereby grown ~40.000 clones turned out to be unequivocally white. These were picked and submitted to the same chromosomal sequencing of the site(s) of insertion as before. Their analysis showed (Figure 3B and Table S2 of Additional File 1) that 6 mutants had mini-Tn*5 *inserted throughout the *lacZ *gene, whereas 12 of them occurred in *xylR*. Since we found 18 different insertions and the length of DNA whose interruption gave the white colony phenotype was about 5 kb, the transposition appeared to occur at gross frequency of ~4 insertions/kb i.e. equivalent to a 4 x coverage of the entire genome (taking an average size of 1 kb/gene). This is surely an underestimation, because the selection procedure on minimal medium avoids the growth of auxotrophic mutants. This is surely the reason why we did not get any insertion in the *rpoN *gene, because such mutants grow poorly in the absence of glutamine [35] and thus fail to form sizable colonies in the minimal medium employed for selection (Additional File 1, Figure S4).

### Exploration of the regulatory landscape of the catabolic *Pu *promoter of *P. putida*

The σ^54^-dependent *Pu *promoter employed above is the principal regulatory element at play in the regulation of a complex system for biodegradation of *m*-xylene in strain *P. putida *mt-2 [36]. *P. putida *MAD1 strain keeps the essential components of the *m*-xylene sensor system, fused to a *lacZ *reporter. The high performance of pBAM1 just described was thus exploited to survey the genome of *P. putida *for genes which could influence -not abolish- *lacZ *output in the hope of identifying novel functions which may well shed some light on the physiological regulation of the *Pu *promoter [37]. To this end, the collection of ~40.000 Km^R ^colonies derived from *P. putida *MAD1 plated on M9-citrate with kanamycin and exposed to *m*-xylene was examined for the appearance of paler blue tones or unusual patterns of Xgal in the otherwise dark blue of the control colonies that peak at the colony centre. Seven of these (Figure 3D and Table S3 of Additional File 1) were chosen for further analysis. The sequence of the corresponding sites of insertion revealed at least two types of genes that influenced the outcome of the *Pu*-*lacZ *reporter. One group is constituted by an insertion in *dnaJ*, which appears to downregulate *Pu *(Figure 3D). DnaJ is a heat-shock protein that stimulates the ATPase activity of DnaK [38] and is perhaps involved in the pathway for proper folding of σ^54 ^(RpoN; [39]). A similar Xgal distribution pattern is observed when the PP1841 gene is disrupted (Figure 3D). Yet, the most unusual phenotype of the *Pu*-*lacZ *fusion carried by *P. putida *MAD1 appeared in an insertion within the intergenic region between *cstA*, a gene, which encodes a carbon-stress response protein [40], and PP4642, a type IV pilus assembly gene. In these cases (Figure 3D), the colonies displayed a double-ring distribution of the dye that suggested an influence of either or both of these proteins in adjusting the physiological control of *Pu *activity [37]. Other interesting phenotypes were produced by mutations in *cysD *and *cysNC *genes, the loss of which produce small, slow-growing colonies with a distinct *fisheye *distribution of Xgal. These mutations are expected to bring about a general deficiency of cysteine [41], which could directly or indirectly affect transcriptional activity (Additional File 1, Table S3). Needless to say, these are preliminary observations that require further examination (see other insertions in Table S3 of Additional File 1). In the meantime, these results illustrate the power of the genetic tool employed for tackling regulatory phenomena.

### Survey and localization of highly-expressed proteins in *Pseudomonas putida*

Although the literature reports many systems for generating fluorescent fusion proteins [42,43] we exploited the layout of the pBAM1 plasmid for constructing a variant able to produce in vivo random insertions of the GFP sequence in chromosomal genes. We reasoned that if a promoterless and leaderless GFP inserts in a gene in the right orientation and in the correct frame we should be able to detect green colonies when insertion occurs either in non essential genes expressed at very high rates or in their permissive termini (note that the final GFP fusions are single-copy). To explore this notion, we constructed a pBAM1 derivative in which the PvuII insert (i.e. the whole mini-transposon part) was replaced by a synthetic DNA with a number of new features. First, the new transposon had the ME-I sequence adjacent to the second codon of a *gfp *variant which is optimized for prokaryotic gene fusions [44] and edited to eliminate a native PvuII site, located at position 41-36 bp from the 3' end of the gene (Figure 2B). In-frame insertions-to-be therefore keep the 5'-end of the truncated gene followed by a 9 amino acid linker resulting from translation of the ME-I mini-transposon end, and completed by the *gfp *gene. Such insertions thus generate hybrid proteins rather than transcriptional fusions, in a way that makes fluorescence to report net gene expression, not only production of mRNA. The second feature of the transposon was the positioning of the Km^R ^cassette (the same as that in pBAM1) downstream of the *gfp *gene, but keeping its own promoter. This ensured that selection for resistance to this antibiotic was independent of orientation and read-through transcription from inserted genes. The thereby refactored pBAM1 derivative was named pBAM1-GFP (Figure 2B; Table 3; GenBank: HQ908072). With this plasmid in hand, we mutagenized *P. putida *KT2440 with the tri-parental mating procedure described above, obtaining the same frequencies than those reported above for pBAM1. Exconjugant clones were allowed to grow to a sizable dimension and inspected for the occurrence of green fluorescent colonies by illuminating the plates with blue light. The frequency of appearance of such strong green fluorescent colonies was 1.17 ± 0.1 × 10^-3^.

**Table 3:** Bacteria and plasmids

Strains	Description/relevant characteristics	Reference
*E. coli*		
CC118λ*pir*	Δ(*ara*-*leu*), *araD*, Δ*lac*X174, *galE*, *galK, phoA, thi1, rpsE, rpoB, argE (Am), recA1*, lysogenic λ*pir*	[4]
HB101	Sm^R ^ , *hsdR*^-^*M*^+^, *pro, leu, thi, recA*	[55]
*P. putida*		
KT2440	mt-2 derivative cured of the TOL plasmid pWW0	[58]
MAD1	KT2440 Rif^R^ , Tel^R^, *xylR*^+^ , *Pu*-*lacZ*	[34]
**Plasmids**		

pRK600	Cm^R^; *ori*ColE1, RK2 *mob^+^, tra^+^*	[15]
pBAM1	Km^R ^Ap^R^; *ori*R6K	This work
pBAM1-GFP	Km^R ^Ap^R^; *ori*R6K, GFP	This work

Rif: Rifampicin; Tel: Tellurite.

**Figure 4:**
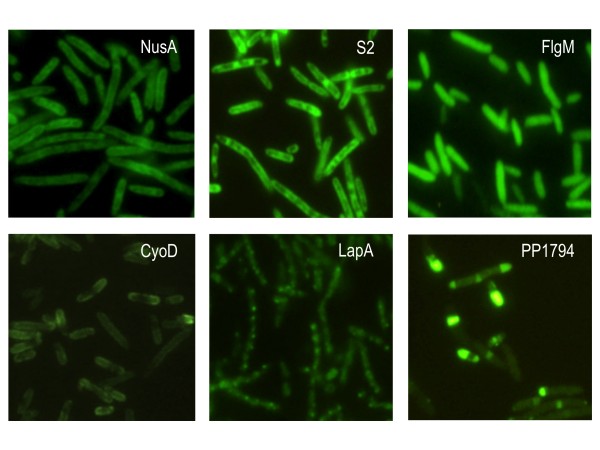
**Subcellular localization of high-fluorescence GFP fusions generated by mutagenesis of *P. putida *with mini-Tn*5*GFPKm**. Cultures of the cells under examination were grown until stationary phase in LB medium and prepared for epifluorescence microscopy as explained in Materials and Methods. The upper panel shows examples of GFP fused to cytosolic proteins, as indicated, whereas in lower panel contains GFP fusions in three different membrane-associated proteins. Table S4 of Additional File 1 provides more details of the GFP fusions generated.

A total 19 clones were picked for further analyses. The sites of insertion were sequenced as before (see Materials and Methods), using ARB6/GFP-extR primers in the first PCR round and ARB2/GFP-intR in the second one, then sequenced with primer GFP-intR (Table 2). 15 insertions were located in different genes. Three independent transpositions were located in the essential gene *rplM*, two of which were identical, whereas the third one mapped in another position within the gene. Finally, two different transpositions were found both in gene PP1794 and *fliC *(for details see Table S4 of Additional File 1). A good share of the GFP fusions were located in genes anticipated to be highly expressed (e.g. ribosomal proteins). Interestingly, such proteins are believed to be essential, indicating that the GFP fusion had occurred in permissive sites that did not affect their functionality. But apart from ribosomal protein genes, we found highly fluorescent insertions in functionally diverse genes (Table S4, Additional File 1). These included transcription factors, cell envelope-related proteins and hypothetical proteins of unknown functions. The location of fluorescent signals in single cells was then investigated in each case by fluorescence microscopy, the most informative results being shown in Fig 4. In most cases, green signals appeared to be somehow localized in specific cell sites or compartments. This was not altogether surprising for proteins known or predicted to be associated with the membrane. CyoD::GFP was clearly bound to the cell contour (more intense at the poles), as would be expected of a protein that forms part of the membrane-bound respiratory chain [45]. LapA::GFP originates in a large loosely surface-associated protein that is exported through an ABC transporter system [46]. That fluorescence appears in this case in regularly spaced foci along the longitudinal cell axis suggests the dots to be the sites of export to the extracellular medium. Yet, the most unusual appearance was that of the PP1794::GFP fusion. This ORF encodes a protein predicted to have a putative outer membrane location. The hybrid product resulting from its fusion to GFP was near entirely confined to the cell poles and displayed a clear-cut boundary with the rest of the cell, an unprecedented behaviour that will be the subject of future studies. Apart of such envelope-related proteins we also found a non-homogenous distribution of GFP in fusions to ribosomal proteins (Figure 4). We believe that these high-fluorescent sites can be related to the so-called *translation factories *that seem to gather most of the ribosomal machinery of individual cells [47]. More unexpected was the high signal brought about by the NusA::GFP fusion. In *E. coli*, this protein is a transcription termination/anti-termination factor that acts either way depending on its association to other cellular proteins [48]. While its high level of expression in *P. putida *was unexpected, its uneven distribution in single cell probably reflected also the occurrence of *transcription factories *[47] in this bacterium. Finally, one FliC::GFP fusion was found to give an uniform GFP signal throughout individual cells. The flagellin protein FliC is the main structural component of the flagella [49]. That *fliC::gfp *cells lacked any swimming motility (data not shown) indicated that the function had been knocked-out. It is hence likely that the FliC::GFP cannot enter the secretion pathway and it freely diffuses in the cytoplasm as a result. However, the FlgM::GFP fusion also originated evenly fluorescent cells (Figure 4), but in this case the transposition did not affect its function since this strain was still motile (not shown).

As discussed before, the selection conditions for the mutagenesis experiment just mentioned were such that they ruled out inactivation of essential and metabolic genes necessary for growth in minimal medium. Also, GFP fusions may conceal the original localization of the inserted protein (as just seen with FliC). However, random generation of fluorescent fusions of the sort discussed above pinpoints proteins that are highly expressed under physiologically relevant conditions. We argue that this may become a phenomenal tool to tackle the still standing question of gene expression sites *vs*. chromosomal localization [50,51], an important issue that is beyond the scope of this paper.

## Conclusion

We have created a synthetic plasmid composed of multiple formatted and optimized functional parts that behave as predicted -both individually and as an integrated system. To the best of our knowledge this is the first report since the early 90s that describes a fully edited genetic tool optimized and streamlined for its final applications -rather than relying on cutting and pasting naturally occurring sequences [52]. In a nutshell, non-functional DNA sequences were trimmed-off, common restriction sites present outside the multiple cloning site inside the mobile element were eliminated and the plasmid was designed following a modular pattern in which each business sequence was flanked by non-frequent restriction sites. In this respect, the key features of pBAM1 include not only the removal of many bottlenecks that flawed utilization of many of its predecessors, but also the incorporation of a fixed standard for physical assembly and exchange, where required, of new DNA pieces while maintaining its overall layout. The modularity of the design and the origin of the parts in mobile elements, which are endowed with considerable orthogonality, enable pBAM1 for two specific applications. The first, straightforward application is the use of pBAM1 as a high-throughput mutational analysis tool [41]. Second, more important, the new vector allows exploitation of the cargo site (Figure 1 and 2) for placing a whole collection of extra genetic *gadgets *for expression of heterologous genes, reporter systems and environmental markers at user's will. This includes the possibility of cloning large DNA fragments inside the mobile element for a final implantation of new traits into the chromosome of the target strain. Given the randomness and the high frequencies of such insertions, one can then select the insertion out of a large collection, which adjusts expression of the desired feature to the right level under the required operation conditions [53,54]. Furthermore, the ease of replacement of the antibiotic resistance marker (or any other functional part) allows the same transposition/delivery system to be reused for subsequent insertions. In sum, this work shows the value of DNA synthesis and standardization of functional modules for combining in a single genetic tool many valuable properties that are otherwise scattered in various vectors and rendered useless for the lack of fixed assembly formats. We anticipate pBAM1 to become one frame of reference for the construction of a large number of vectors aimed at deployment of heavily engineered genetic and metabolic circuits.

## Methods

### Strains, plasmids and media

The bacterial strains and plasmids used in this study are listed in Table 3. Bacteria were grown routinely in LB (10 g l^-1 ^of tryptone, 5 g l^-1 ^of yeast extract and 5 g l^-1 ^of NaCl). *E. coli *cells were grown at 37°C while *P. putida *was cultured at 30°C. Selection of *P. putida *cells was made onto M9 minimal medium plates [55] with citrate (2 g l^-1^) as the sole carbon source. Antibiotics, when needed, were added at the following final concentration: ampicillin (Ap) 150 μg ml^-1 ^for *E. coli *and 500 μg ml^-1 ^*for P. putida*, kanamycin (Km) 50 μg ml^-1 ^and chloramphenicol (Cm) 30 μg ml^-1 ^for both species. 5-bromo-4-chloro-3-indolyl- β-D-galactopyranoside (Xgal) was added when required at 40 μg ml^-1^. The *Pu-lacZ *fusion of *P. putida *MAD1 (Table 3) was induced by exposing cells to saturating *m*-xylene vapors.

### DNA techniques

Standard procedures were employed for manipulation of DNA [55]. Plasmid DNA was prepared using Wizard Plus SV Minipreps (Promega) and PCR-amplified DNA purified with NucleoSpin Extract II (MN). Oligonucleotides were purchased from SIGMA. For colony PCR a fresh single colony was picked from a plate and transferred directly into the PCR reaction tube. Transposon insertions were localized by arbitrary PCR of genomic DNA [33]. Single colonies were used as the source of the DNA template for the first PCR round, which was programmed as follows: 5 minutes at 95°C, 6 cycles of 30 s at 95°C, 30 sec at 30°C, and 1 min and 30 s at 72°C; 30 cycles of 30 s at 95°C, 30 s at 30°C and 1 min and 30 s at 72°C. This was followed by an extra extension period of 4 min at 72°C. The primers used for the first round included ARB6 in combination with either ME-O-extF or ME-I-extR/GFP-extR (described in Table 2). 1 μl of the resulting product was then used as template for the second PCR round, using with the following conditions: 1 min at 95°C, 30 cycles of 30 s at 95°C, 30 sec at 52°C and 1 min and 30 sec at 72°C, followed by an extra extension period of 4 min at 72°C. The second round was performed with ARB2 and ME-O-intF or ME-I-intR/GFP-intR (Table 2). PCR reaction mixtures were purified and sequenced with either ME-O-intF or ME-I-intR/GFP-intR primers. DNA sequences were visually inspected for errors and analyzed using the *Pseudomonas *Genome Database_v2 _(http://www.pseudomonas.com) and blast (http://blast.ncbi.nlm.nih.gov/Blast.cgi) to map the precise transposon insertion point. To ascertain the conservation level of the 9-bp target sequence of Tn*5 *transposase we made use of WebLogo 3, a web based application available at http://weblogo.berkeley.edu/logo.cgi[56]. DNA synthesis was outsourced from Geneart (http://www.geneart.com). The nucleotide sequences of the pBAM1 and pBAM1-GFP plasmids were submitted to the GenBank database (http://www.ncbi.nlm.nih.gov/genbank/) under the corresponding accession numbers HQ908071 and HQ908072.

### Suicide delivery of mini-transposons

pBAM1 and its derivatives were entered into target cells by either mating or electroporation. In the first case, the plasmid was mobilized from *E. coli *CC118λ*pir *(pBAM1) donor cells into *Pseudomonas putida *(KT2440 or MAD1 strains, Table 3) with the assistance of the helper strain *E. coli *HB101 (pRK600). To this end, cells were grown overnight with the appropriate antibiotics. Cells were washed with 1.0 ml of 10 mM MgSO_4 _and mixed in 1:1:1 ratio into 5 ml of 10 mM MgSO_4 _solution to obtain a final OD_600 _of 0.03 (3 × 10^7 ^cells) of each strain. Then, the tri-parental mating mixture was concentrated and laid onto a Millipore filter disk (0.45 μm pore-size, 13-mm diameter). The filters were incubated at 30°C onto the surface of LB agar plates. At the desired incubation time, the filter was transferred to a 5 ml of a 10 mM MgSO_4 _solution and vortexed to re-suspend the cells. Afterwards, appropriate dilutions were plated onto adequate selective medium as indicated for counter-selecting the donor cells in the mating. Alternatively, *P. putida *electrocompetent cells were prepared following the protocol described in [57]. In this case, 100 ng - 500 ng of pBAM1 plasmid DNA were added to a 100 μl aliquot suspension containing a total of 6 × 10^10 ^cells. The mixture was then transferred into a 2 mm gap width cuvette and electroporated with the settings of a single pulse of 2.5 kV (field strength of 12.5 kV cm^-1^) with a time constant of ~5 msec using program EC2 in a MicroPulser™ (BioRad). Following electropulsing, cells were quickly supplemented with 1 ml of LB and incubated at 30°C for 1 h. Then, adequate dilutions of such a suspension were plated onto M9-citrate medium plus Km for selection of mini-transposon insertions. Whether from conjugation or from electroporation, Km^R ^clones were streaked out, single colonies checked for the loss of the plasmid marker (Ap^R^), and the genomic DNA adjacent to the sites of insertion sequenced as explained above.

### Fluorescence detection methods

Bacterial colonies growing on agar plates were inspected for emission of green fluorescence born by GFP by illumination with a 470 nm light (Safe Imager™ blue light transilluminator, Invitrogen). For visualization of GFP in individual bacteria, *P. putida *cells were grown up to stationary phase either in minimal M9-citrate medium or in LB. 12 ml of the cultures diluted to an OD_600 _of 0.5 were applied to a poly-L-Lysine-padded microscope slide and covered with mounting media for fluorescence *Vectashield *(Vector laboratories Inc.). Preparations were imaged with an Olympus BX61 microscope. Pictures were taken with a 100x immersion oil lens and an Olympus U-MNIBA2 filter (excitation filter 470/20 nm, emission filter 515/35 nm, beam splitter 505LP) to record fluorescence signals.

## Authors' contributions

VdL planned and coordinated the research project. VdL, EMG and BC conceived and designed the experiments. EMG performed the pBAM1 characterization while BC constructed and implemented the pBAM1-GFP plasmid. MAR streamlined the design of the different modules of the pBAM1 plasmid. All authors have read and approved the manuscript.

## Supplementary Material

Additional File 1 **Supplementary Figures and Tables**. Figure S1: Transposition time course during conjugative delivery of mini-Tn*5 *Km from pBAM1. Figure S2: Mini-Tn*5 *Km insertion mapping example. Figure S3: Consensus insertion site of the mini-Tn*5 *Km of pBAM1 in the genome of *P. putida*. Figure S4: Growth of *P. putida *wild type and an *rpoN *mutant strain in minimal medium. Table S1: Localization of mini-Tn*5 *Km transposon insertions within the *P. putida *KT2440 genome. Table S2: Details of the sites of insertion of mini-Tn*5 *Km in *P. putida *MAD1 *white *mutants. Table S3: Details of the sites of insertion of mini-Tn*5 *Km in *P. putida *MAD1 producing unusual *white/blue *patterns in X-gal plates. Table S4: Location of GFP-fusions generated with pBAM1-GFP within the *P. putida *KT2440 genome. Click here for file
