# Supplementary material for: pBAM1: an all-synthetic genetic tool for analysis and construction of complex bacterial phenotypes
Source: BMC Microbiol. 2011 Feb 22;11:38. doi: 10.1186/1471-2180-11-38 (PMC3056738; doi:10.1186/1471-2180-11-38)
Supplement: Additional File 1 — Supplementary Figures and Tables. Figure S1: Transposition time course during conjugative delivery of mini-Tn5 Km from pBAM1. Figure S2: Mini-Tn5 Km insertion mapping example. Figure S3: Consensus insertion site of the mini-Tn5 Km of pBAM1 in the genome of P. putida. Figure S4: Growth of P. putida wild type and an rpoN mutant strain in minimal medium. Table S1: Localization of mini-Tn5 Km transposon insertions within the P. putida KT2440 genome. Table S2: Details of the sites of insertion of mini-Tn5 Km in P. putida MAD1 white mutants. Table S3: Details of the sites of insertion of mini-Tn5 Km in P. putida MAD1 producing unusual white/blue patterns in X-gal plates. Table S4: Location of GFP-fusions generated with pBAM1-GFP within the P. putida KT2440 genome. [file 1471-2180-11-38-S1.PDF]

Additional File 1

pBAM1: an all-synthetic genetic tool for analysis and construction of complex bacterial phenotypes

Esteban Martínez-García[1], Belén Calles[1], Miguel Arévalo-Rodríguez[2] and Víctor de Lorenzo[1]

[1]Systems Biology Program. Centro Nacional de Biotecnología-CSIC, Campus de Cantoblanco, 28049, Madrid (Spain). [2]Biomedal SL, 41092, Sevilla (Spain).

Figure S1. Transposition time course during conjugative delivery of mini-Tn*5* Km from pBAM1.

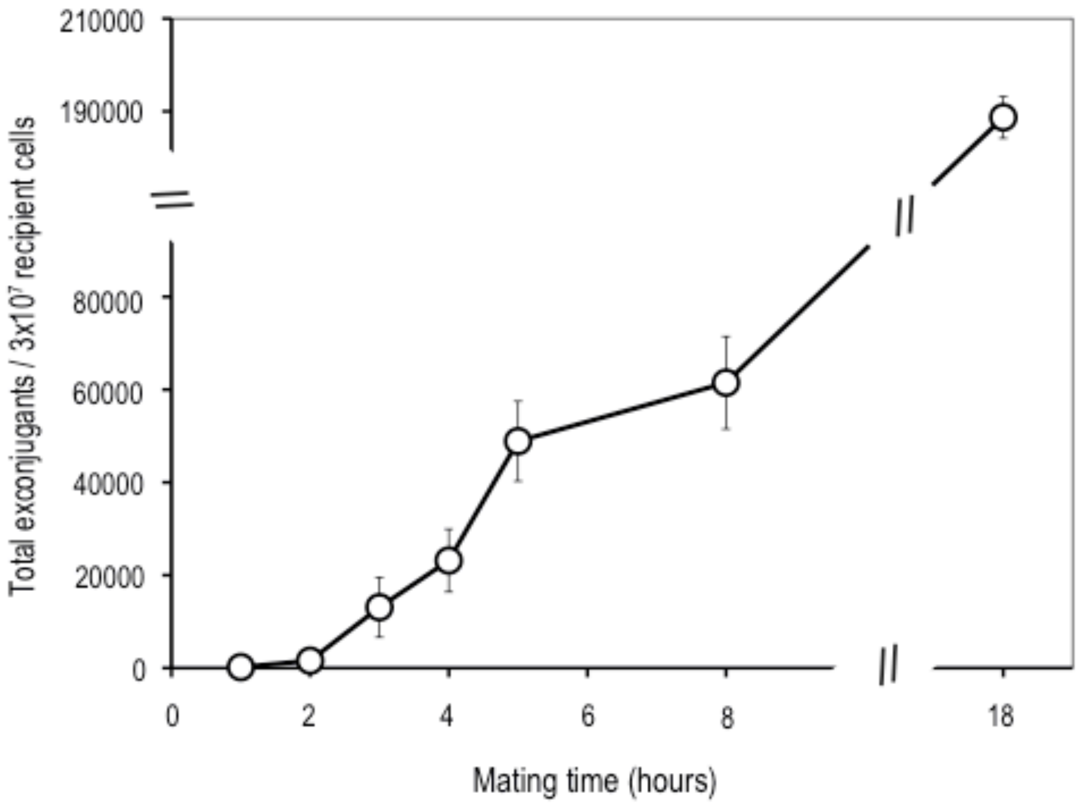

Tri-parental matings were employed for suicide delivery of pBAM1. At the times indicated the filters were collected, cells re-suspended, and appropriate dilutions plated onto selective media. The number of kanamycin resistant colonies was determined for each time point. The average of three independent experiments with their standard deviation is shown.

Figure S2. Mini-Tn*5* Km insertion mapping example

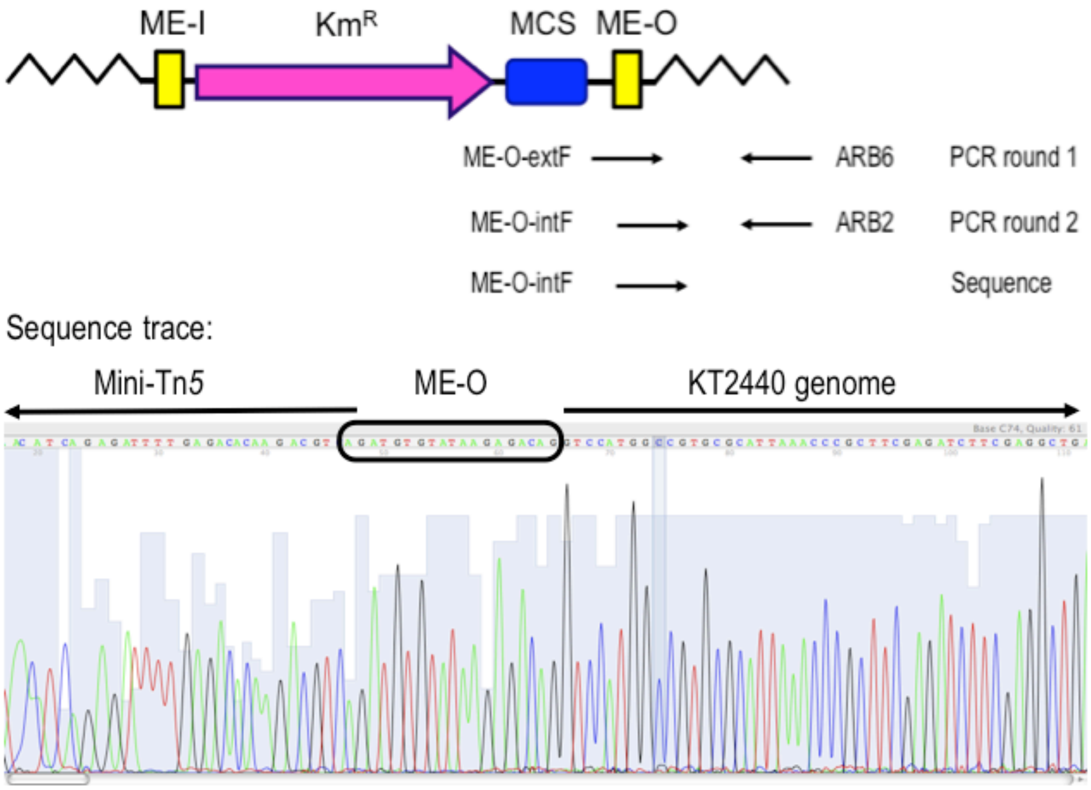

Schematic representation of the locations of ME-O and arbitrary primers employed in the two rounds of arbitrary PCR [1], and, consequently, the ME-O primer used for sequencing. Below, a fragment of a typical chromatogram showing the base calling that enabled the identification of the junction point between the ME-O (circled) and the *P. putida* KT2440 genome.

Figure S3. Consensus insertion site of the mini-Tn*5* Km of pBAM1 in the genome of *P. putida*

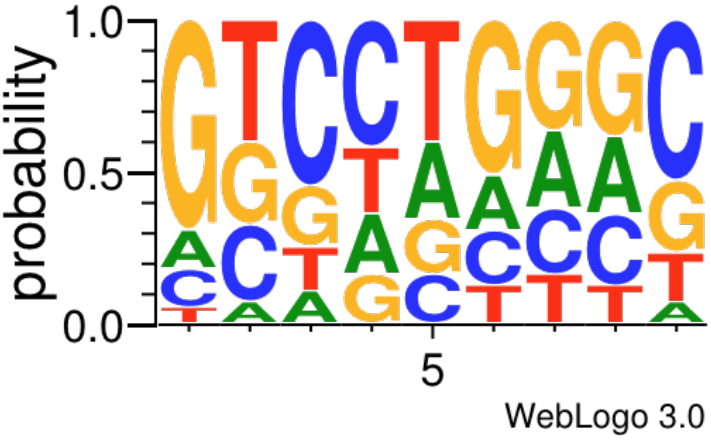

A total of fifty-five junction sequences were analyzed to create a DNA logo sequence using WebLogo 3 software (http://weblogo.threeplusone.com/; [2]. The probability of having a specific nucleotide base in a determined position is shown. In this calculation we corrected for the GC content of *P. putida* KT2440 (GC% 61.5).

Figure S4. Growth of *P. putida* wild type and an *rpoN* mutant strain in minimal medium.

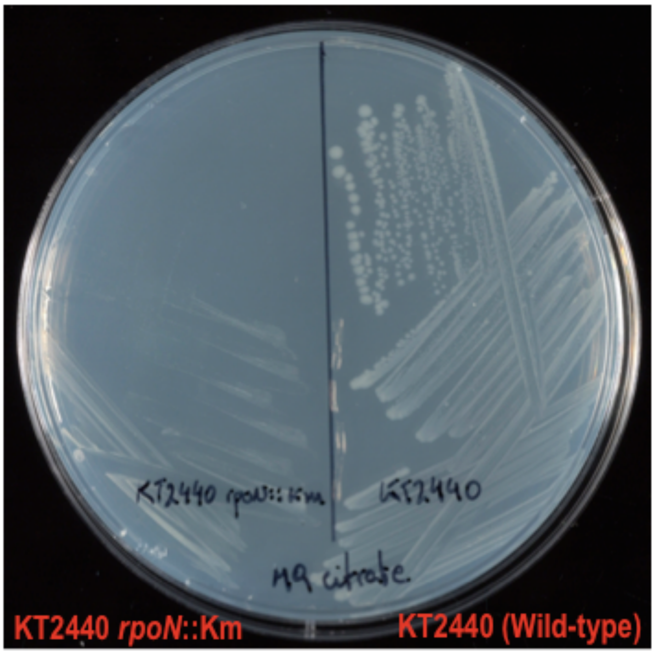

This picture illustrates the lack of significant growth of the *rpoN* mutant (left) of *P. putida* in M9 minimal medium with citrate after several days of incubation at 30 ºC. The control wild-type strain is streaked out to the right.

Table S1. Localization of mini-Tn*5* Km transposon insertions within the *P. putida* KT2440 genome[a]

ME-O-intF							
				Coordinates in the PCR fragment		Coordinates in KT2440 genome	
**Mutant #**	**PP#**	**Gene**	**PCR fragment Length**	**pBAM1 sequence read**	**KT2440 genome read**	**start**	**nt % identity**
1	4789		640	1-10 bp	11-640	5451061	99.5
2	4008	*clpA*	789	1-24 bp	25-789	4518079	100
10	intergenic-(0656)		561	1-31 bp	32-561	763691	92
11	intergenic-(0021)		407	1-41 bp	42-407	25712	96
12	0488		587	1-33 bp	34-587	574607	99
13	4108		781	1-33 bp	34-781	4643969	100
14	intergenic-(1628)		553	1-37 bp	38-553	1826516	97
18	2772		295	1-21 bp	22-295	3156380	99.6
19	4936	*waaL*	351	1-23 bp	24-351	5617837	100
20	5342		257	1-21 bp	22-257	6090024	100
21	4358	*fliM*	479	1-40 bp	41-479	4950154	96
22	3378	*proC-1*	271	1-40 bp	41-271	4306289	100

ME-I-intR							
				Coordinates in the PCR fragment		Coordinates in KT2440 genome	
**Mutant #**	**PP#**	**Gene**	**PCR fragment Length**	**pBAM1 sequence read**	**KT2440 genome read**	**start**	**nt % identity**
1	4789		122	1-45 bp	46-122	5451053	100
2	4008	*clpA*	607	1-51 bp	52-607	4518071	99
10	intergenic-(0656)		530	1-42 bp	43-530	763683	100
11	intergenic-(0021)		551	1-42 bp	43-551	25704	100
12	0488		1023	1-45 bp	46-1023	574615	98
13	4108		571	1-44 bp	45-571	4643977	100
14	intergenic-(1628)		310	1-38 bp	39-310	1826508	100
18	2772		836	1-52 bp	53-836	3156372	97
19	4936	*waaL*	788	1-42 bp	43-788	5617829	99.8
20	5342		551	1-45 bp	46-551	6090016	94
21	4358	*fliM*	840	1-45 bp	46-840	4950146	100
22	3778	*proC-1*	447	1-45 bp	46-447	4306297	100

[a] Both ends of the mini-Tn*5* Km were employed to locate each of the transposons in the genome of the 12 mutants. The Table shows: the length of the PCR amplicon obtained in the arbitrary PCR, the number of nucleotides from the PCR that match the end of the mini-transposon, the PCR fragment length that reads into the KT2440 genome, with its corresponding genome coordinates and identity percentage at nucleotide level. The nt identity is not 100% due to some minor sequence ambiguities in the PCR fragments.

Table S2. Details of the sites of insertion of mini-Tn*5* Km in *P. putida* MAD1 *white* mutants[a].

			Coordinates in the PCR fragment			
**MAD1 mutant #**	**Gene**	**PCR fragment Length**	**pBAM1 sequence read**	**MAD1 genome read**	**Position**	**nt % identity**
**1**	*xylR*	937	1-33 bp	34-937	26	96
**2**	*xylR*	1012	1-34 bp	35-739	704	99
**4**	*xylR*	994	1-33 bp	34-462	462	99
**5**	*xylR*	762	1-35 bp	36-762	461	98
**10**	*xylR*	689	1-36 bp	38-689	1691	95
**12**	*xylR*	948	1-33 bp	34-396	130	96
**14**	*xylR*	366	1-28 bp	29-338	984	98
**27**	*xylR*	845	1-42 bp	43-845	1584	90
**40**	*xylR*	847	1-53 bp	54-847	794	98
**46**	*xylR*	485	1-40 bp	41-485	1326	99
**61**	*xylR*	953	1-37 bp	38-953	1345	94
**62**	*xylR*	767	1-33 bp	34-767	1303	91
**6**	*lacZ*	874	1-34 bp	35-874	1537	97
**11**	*lacZ*	540	1-38 bp	39-540	1972	92
**42**	*lacZ*	866	1-20 bp	21-866	2086	97
**52**	*lacZ*	622	1-34 bp	35-182	148	85
**57**	*lacZ*	560	1-37 bp	38-560	1419	99
**63**	*lacZ*	985	1-53 bp	54-985	1196	97

[a] Arbitrary PCR followed by sequencing with the primers indicated in the text was used to identify the sites where insertion had occurred. The length of the PCR amplicon obtained in the arbitrary PCR is indicated in each case along with the number of nucleotides from the PCR that match the end of the mini-transposon. The Table indicates as well the length of the PCR fragment that reads into the *P. putida* MAD1 genome, the position relative to the start codon of the corresponding gene, and the identity percentage of the PCR fragment at the nucleotide level (<100% due to sequence ambiguities in the PCR fragments).

Table S3. Details of the sites of insertion of mini-Tn*5* Km in *P. putida* MAD1 producing unusual *white/blue* patterns in X-gal plates [a].

					Coordinates in the PCR fragment		Coordinates in MAD1 genome	
**MAD1 mutant #**	**Gene**	**PP#**	**Putative function**	**PCR fragment Length**	**pBAM1 sequence read**	**MAD1 genome read**	**Position**	**nt % identity**
3	*cysNC*	1304	Sulfate adenylyltransferase subunit 1	689	1-53 bp	54-689	1492082	94
59	*cysNC*	1304	Sulfate adenylyltransferase subunit 1	1030	1-42 bp	43-1030	1490835	98
19	*cysD*	1303	Sulfate adenylyltransferase subunit 2	922	1-32 bp	33-922	1489869	97
30	intergenic-*cstA*-PP4642			841	1-31 bp	32-841	5266758	98
56		1946	Short chain dehydrogenase	739	1-32 bp	33-739	2202115	100
58	*dnaJ*	4726	Chaperone protein DnaJ	632	1-34 bp	35-632	5375412	98
60		1841	Cytochrome c family protein	969	1-42 bp	43-969	2064886	97

[a] The Table shows the gene/region inserted by the transposon, the corresponding PP number, the putative function of the inactivated gene, the length of the PCR amplicon obtained in the arbitrary PCR, the number of nucleotides that match the end of the mini-transposon, the length of the PCR fragment that reads into the *P. putida* MAD1 genome, the genome coordinates were mini-Tn*5* Km is located, and the identity percentage of the PCR fragment at nucleotide level (<100% due to sequence ambiguities in the PCR fragments).

Table S4. Location of GFP-fusions generated with pBAM1-GFP within the *P. putida* KT2440 genome[a]

Mutant #	PP#	Gene	Function	length (aa)	Insertion region
1	4493		Putative oxidase	1006	K603
2	1794		Hypothetical protein, putative OM	480	D432
3	4713	*nusA*	Transcriptional elongation factor	493	L437
4	1911	*rpmF*	Ribosomal protein L32	60	D14
5	4709	*rpsO*	Ribosomal protein S15	107	Y96
6	4378	*fliC*	Structural component of flagella	687	D269
7	5139	*cadA2*	Metal iron translocating ATPase	750	A608
8	1591	*rpsB*	Ribosomal protein S2	245	A239
9	1315	*rplM*	Ribosomal protein L13	142	Q136
10	1315	*rplM*	Ribosomal protein L13	142	H132
11	0274		HTH domain protein	179	E107
12	4395	*flgM*	Anti sigma factor FlgM	104	A90
13	0721	*rplM*	Ribosomal 5S rRNA E-loop binding Ctc/L25/TL5	217	K121
14	4378	*fliC*	Structural component of flagella	687	D469
15	0411		Polyamine ABC transporter	374	S305
16	1794		Hypothetical protein, putative OM	480	D437
17	0168	*lapA*	Surface-associated protein	8682	ND
18	0815	*cyoD*	Ubiquinol oxidase (IV)	110	D70

[a] Arbitrary PCR followed by sequencing with the GFP-intR primer was used to identify the proteins where the fusion had occurred. PP# indicates the *P. putida* KT2440 locus number. The gene name (when known) is indicated along with the putative function of the protein, the number of amino acids in its primary sequence and the GFP insertion point. ND: not determined due to the presence of a large number of internal repeats within the sequence.

REFERENCES

1. Das S, Noe JC, Paik S, Kitten T: An improved arbitrary primed PCR method for rapid characterization of transposon insertion sites. *J Microbiol Methods* 2005, 63:89-94.

2. Crooks GE, Hon G, Chandonia JM, Brenner SE: WebLogo: a sequence logo generator. *Genome Res* 2004, 14:1188-1190.
